# Preliminary effectiveness of breast cancer screening among 1.22 million Chinese females and different cancer patterns between urban and rural women

**DOI:** 10.1038/srep39459

**Published:** 2016-12-20

**Authors:** Yubei Huang, Hongji Dai, Fengju Song, Haixin Li, Ye Yan, Zhenhua Yang, Zhaoxiang Ye, Sheng Zhang, Hong Liu, Yali Cao, Li Xiong, Yahong Luo, Tie Pan, Xiangjun Ma, Jie Wang, Xiuling Song, Ling Leng, Yeping Zhang, Jie Sun, Jialin Wang, Hengmin Ma, Lingzhi Kong, Zhenglong Lei, Yaogang Wang, Wang Peishan, Jiali Han, Xishan Hao, Kexin Chen

**Affiliations:** 1 Department of Epidemiology and Biostatistics, Key Laboratory of Cancer Prevention and Therapy, Tianjin, Key Laboratory of Breast Cancer Prevention and Therapy, Ministry of Education, National Clinical Research Center for Cancer, Tianjin Medical University Cancer Institute and Hospital, Tianjin, China; 2 Department of Radiology, Key Laboratory of Cancer Prevention and Therapy, Tianjin, Key Laboratory of Breast Cancer Prevention and Therapy, Ministry of Education, National Clinical Research Center for Cancer, Tianjin Medical University Cancer Institute and Hospital, Tianjin, China; 3 The 3rd Department of Breast Cancer, Key Laboratory of Cancer Prevention and Therapy, Tianjin, Key Laboratory of Breast Cancer Prevention and Therapy, Ministry of Education, National Clinical Research Center for Cancer, Tianjin Medical University Cancer Institute and Hospital, Tianjin, China; 4 The 2^nd^ Department of Breast Cancer, Key Laboratory of Cancer Prevention and Therapy, Tianjin, Key Laboratory of Breast Cancer Prevention and Therapy, Ministry of Education, National Clinical Research Center for Cancer, Tianjin Medical University Cancer Institute and Hospital, Tianjin, China; 5 Prevention and Cure Center of Breast Disease, Third Hospital of Nanchang, Nanchang, China; 6 Department of Medical Image, Liaoning Cancer Institute and Hospital, Shenyang, China; 7 Department of Cancer Prevention and Control, Liaoning Cancer Institute and Hospital, Shenyang, China; 8 Center for Breast Disease, Haidian Maternal and Child Health Hospital, Beijing, China; 9 People’s Hospital of Feicheng City, Taian, China; 10 Shandong Tumor Hospital and Institute, Jinan, China; 11 Chinese Ministry of Health, Beijing, China; 12 Department of Health Service Management, School of Public Health, Tianjin Medical University, Tianjin, China; 13 Department of Epidemiology, Fairbanks School of Public Health, Simon Cancer Center, Indiana University, Indianapolis, Indiana, USA; 14 Chinese Anti-Cancer Association, Tianjin, China

## Abstract

To determine the preliminary effectiveness of breast cancer screening among Chinese females, 1226714 women aged 35–69 years first received clinical breast examinations. Urban women with suspected cancer received mammography followed by breast ultrasound (BUS), while rural suspected women underwent BUS followed by mammography. After one-year follow-up, 223 and 431 breast cancers were detected among urban and rural women (respectively), with overall detection rates of 0.56/1000 and 0.52/1000. Higher detection rates were significantly associated with older age at screening for both urban and rural women; additionally, urban women were at significantly higher risk if they had no job, no insurance, or were obese; additional risk factors specific to rural women included Han nationality, higher income, being unmarried, and having a family history of cancer (all P values < 0.05). Among screening-detected breast cancers in urban vs. rural women, 46.2% and 38.8% (respectively) were early stage, 62.5% and 66.3% were ≤2 centimeters, 38.0% and 47.3% included lymph-node involvement, and 14.0% and 6.0% were identified as carcinoma *in situ*. All abovementioned cancer characteristics were significantly better than clinic-detected cancers (all P values < 0.001). In conclusion, several important differences were found between urban and rural women in screening effectiveness and patterns of cancer distribution.

The incidence of breast cancer is much lower in China (32.4/100000) than in western countries^1^,^2^. However, incidence has increased by 3–5% annually in both urban and rural areas in China for the last 20 years, much faster than the world average annual increase of 0.5%^3^,^4^. Moreover, the five-year relative survival for breast cancer among Chinese women was only 73.0% in 2003–2005, but 91% in American women in 2005–2011^2^,^5^. Besides the differences in treatment practices between the two countries^1^,^2^,^6^, another important cause for the substantial differences in survival has to do with the stage at which the cancer is detected, which is largely influenced by cancer screening practices.

Although population-based breast cancer screening is widely practiced around the world, especially in developed countries, the coverage of mammography screening for breast cancer is still very low in China. Barriers to the implementation of a population-based mammography screening program include insufficient cost-effectiveness data; the large, widely dispersed population; shortage of mammography equipment; and inadequate insurance coverage for such a program^6^. As a result, the overall opportunistic mammography screening rate in China was only 21.7% in 2010^7^, while the population-based mammography screening in America women was 68.5% in 2008^8^. Moreover, less than 1% of breast cancer patients in China were detected as carcinoma *in situ* at diagnosis in 1999–2008^9^, while in the United States the proportion was 20% among both non-Hispanic white and African American women in 2013^10^.

To raise awareness and knowledge regarding breast cancer among Chinese women, and to collect baseline data for developing feasible breast cancer screening schemes for Chinese urban and rural women, in 2008 the Chinese Ministry of Health launched the Chinese National Breast Cancer Screening Program (CNBCSP) covering 29 provinces. Here we aim to evaluate the preliminary effectiveness of this population-based breast cancer screening program.

## Results

### Demographic Characteristics of CNBCSP

Among urban women in the study, 96.6% were of Han ethnicity, 79.3% had an education level higher than primary school, 94.7% had a job, 72.7% had a family income <3,000 Chinese RMB/month, 67.7% had lived with 1–3 family members, 80.4% had health insurance, 95.8% were being married (excluded single, divorced, separated, or widowed), 70.3% had married at age ≤25 years, 65.8% had no family history of cancer, and 77.9% had a body mass index (BMI) <25 kg/m^2^. Similar distributions of demographic factors were also found among Chinese rural women. However, only 51.9% of rural women had an education level higher than primary school and only 20.6% held jobs, but 60.6% lived with more family members (≥4) (Table 1).

### Overall detection rates of breast cancer

**Figure 1:**
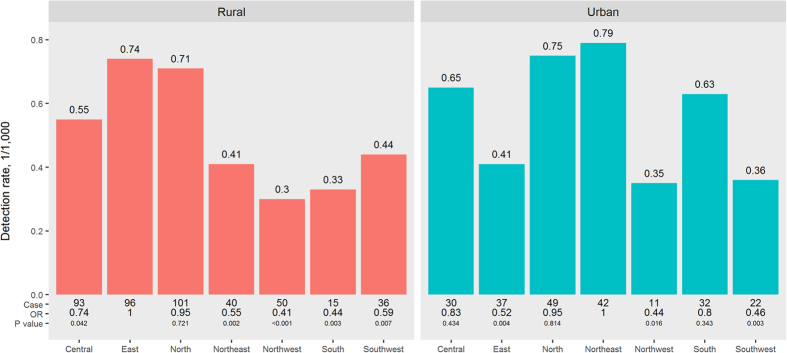
Regional detection rates (1/1000) of breast cancer for urban and rural women from the Chinese National Breast Cancer Screening Program. The central region included Hubei, Hunan, Henan, and Jiangxi; the east included Shandong, Jiangsu, Anhui, Zhejiang, Fujian, and Shanghai; the north included Beijing, Tianjin, Hebei, Shanxi, and Inner Mongolia; the northeast include Liaoning, Jilin, and Heilongjiang; the northwest included Ningxia, Xinjiang, Qinghai, Shaanxi, and Gansu; the south including Guangdong, Guangxi, and Hainan; and the southwest included Sichuan, Yunnan, Guizhou, and Chongqing.

A total of 223 and 431 breast cancers were detected among Chinese urban and rural women (respectively), with overall detection rates of 0.56/1,000 and 0.52/1,000. For urban women, although there was no significant differences in detection rates among the northeast (0.79/1,000), the north (0.75/1,000), the central (0.65/1,000), and the south (0.63/1,000), the detection rate in the northeast was significantly higher than that in the east (0.41/1,000, P = 0.004), the southwest (0.36/1,000, P = 0.003), and the northwest (0.35/1,000, P = 0.016). Among rural women, there was no significant difference between detection rates in the east (0.74/1,000) and those in the north (0.71/1,000). However, the detection rate in the east was significantly higher than that in central (0.55/1,000, P = 0.042), southwest (0.44/1,000, P = 0.0007), northeast (0.41/1,000, P = 0.002), southern (0.33/1,000, P = 0.003), or northwestern China (0.30/1,000, P < 0.001) (Fig. 1). Provincial detection rates of breast cancer for Chinese urban women and rural women are mapped in Supplementary Fig. 1.

### Detection rates of breast cancer among urban women stratified by demographic characteristics

**Figure 2:**
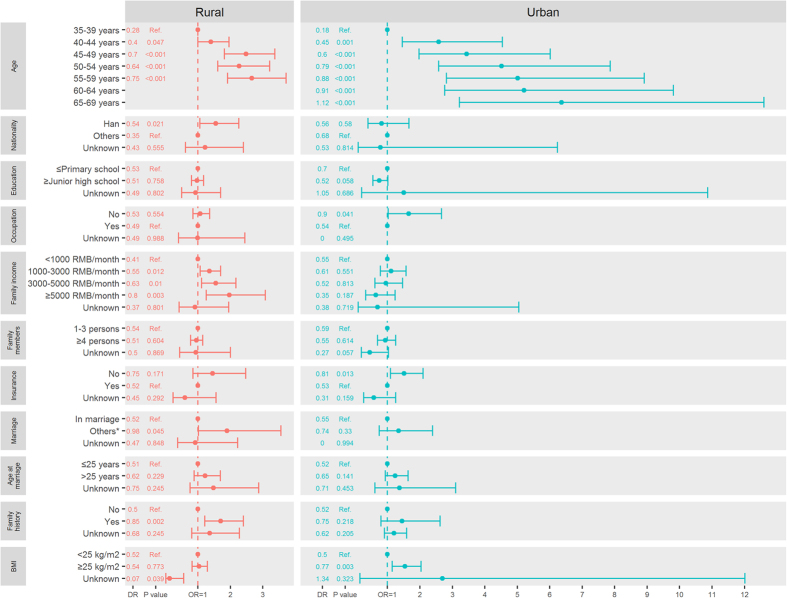
Detection rates (DR, 1/1000) and odds ratios (OR) of breast cancer according to demographic characteristics from the Chinese National Breast Cancer Screening Program. *Included single, divorced, separated, and widowed women. BMI, body mass index.

Among urban women, breast cancer detection rates were significantly higher among women who were older at screening [65–69 years vs. 35–39 years, detection rates: 1.12/1000 vs. 0.18/1000; odds ratio (OR) and its 95% confidential intervals (95% CIs): 6.36 (3.22–12.60), P < 0.001], unemployed women [no occupation vs. with occupation, detection rates: 0.90/1000 vs. 0.54/1000; OR (95% CIs): 1.65 (1.02–2.67), P = 0.041], uninsured women [with insurance vs. no insurance, detection rates: 0.81/1000 vs. 0.53/1000; OR (95% CIs): 1.51 (1.09–2.10), P = 0.013], and those with BMI ≥25 kg/m^2^ [yes vs. no, detection rates: 0.77/1000 vs. 0.50/1000; OR (95% CIs): 1.54 (1.15–2.06), P = 0.003] (Fig. 2, Supplementary Table 1).

**Figure 3:**
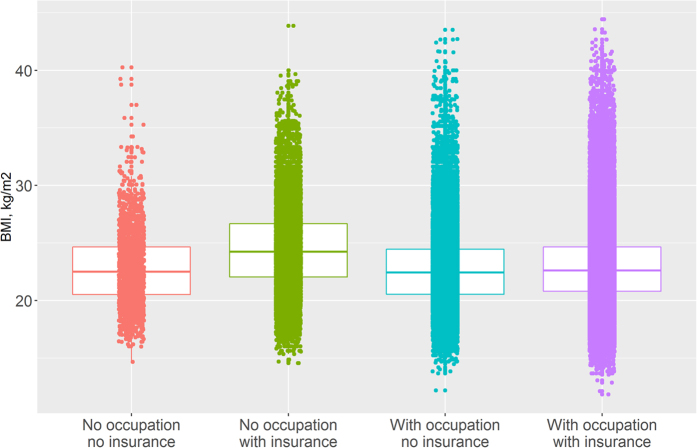
Distribution of body mass index (BMI, kg/m^2^) according to occupation and insurance among Chinese urban women.

The Chi-square test showed that the distribution of occupation was significantly different according to insurance among urban women (P < 0.001, Supplementary Table 2). After reclassifying urban women into four groups according to occupation and insurance (defined as occupation/insurance), we found that the distribution of obesity was significantly associated with occupation/insurance (Fig. 3). In addition, women who had no job but did have insurance were significantly more likely to be obese than other women (P < 0.001, Supplementary Table 3).

**Figure 4:**
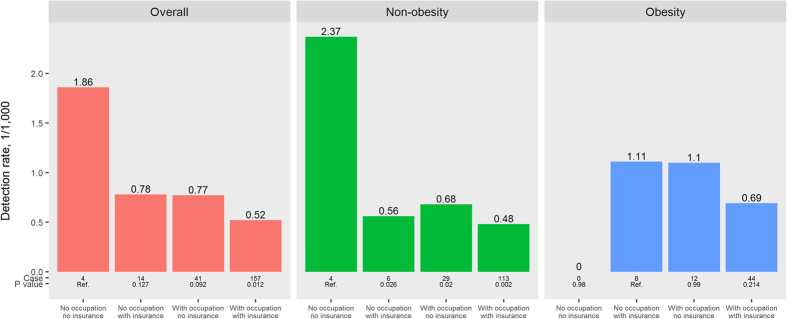
Detection rates (DR, 1/1000) of breast cancer according to occupation/insurance and obesity (body mass index ≥25 kg/m^2^) among Chinese urban women. Data with missing values for occupation, insurance, or body mass index were excluded.

Women who had neither occupation nor insurance had a higher detection rate of breast cancer (1.86/1000) than women with both occupation and insurance (0.52/1000, P = 0.012). After further stratification according to obesity, women with neither occupation nor insurance also had the highest detection rate of breast cancer (2.37/1000) among non-obese women (all P < 0.05). However, there was no significant difference among the four groups of women among obese women (Fig. 4).

### Detection rates of breast cancer among rural women stratified by demographic characteristics

As was the case with urban women, higher detection rates of breast cancer were also significantly associated with elder age at screening among rural women [55–59 years vs. 35–39 years, detection rates: 0.75/1000 vs. 0.28/1000; OR (95% CIs): 2.66 (1.91–3.72), P < 0.001]. Other significant risk factors for rural women included Han ethnicity [others vs. Han, detection rates: 0.54/1000 vs. 0.35/1000; OR (95% CIs): 1.55 (1.06–2.26), P = 0.021], higher family income [≥5000 RMB/month vs. < 1000 RMB/month, detection rates: 0.80/1000 vs. 0.41/1000; OR (95% CIs): 1.97 (1.26–3.08), P = 0.003], being unmarried [included single, divorced, separated, and widowed), detection rates: 0.98/1000 vs. 0.52/1000; OR (95% CIs): 1.90 (1.02–3.56), P = 0.045], and a family history of any kind of cancer [yes vs. no, detection rates: 0.85/1000 vs. 0.50/1000; OR (95% CIs): 1.70 (1.21–2.40), P = 0.002] (Fig. 2, Supplementary Table 1).

The Chi-square test showed that the distribution of family income was significantly associated with marital status among rural women (P < 0.001, Supplementary Table 4). After reclassifying urban women into four groups according to family income and marriage (defined as income/marriage), we also found that the distribution of family history of cancer was significantly associated with income/marriage (P < 0.001, Supplementary Table 5).

**Figure 5:**
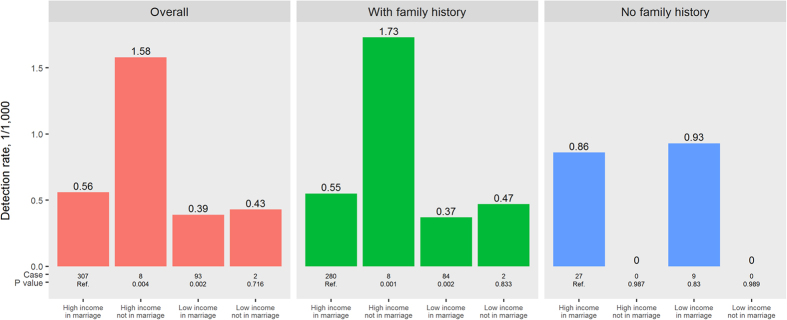
Detection rates (DR, 1/1000) of breast cancer according to family income/marriage and family history (FH) of cancer among Chinese rural women. Data with missing values for family income, marriage, and family history of cancer were excluded. Low income was defined as family income <1000 RMB/month, and high income as family income ≥1000 RMB/month.

The detection rate of breast cancer among married women with high income (0.56/1000) was significantly lower than that among unmarried women with high income (1.58/1000, P = 0.004) but significantly higher than that among married women with low income (0.39/1000, P = 0.002). After further stratification according to family history of cancer, similar results were found among women with a family history of cancer; however, there was no significant difference among the four groups of women without family history of cancer (Fig. 5).

### Tumor characteristics of screening-detected breast cancers

**Figure 6:**
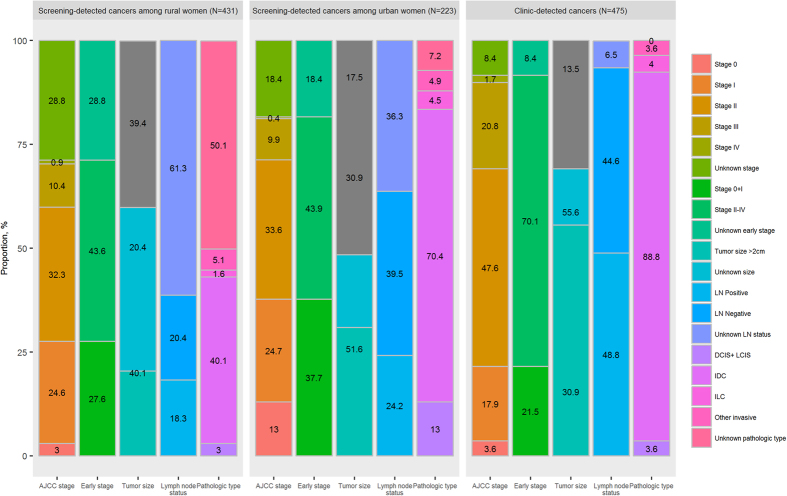
Tumor characteristics of breast cancer detected in the Chinese National Breast Cancer Screening Program and breast cancers detected from clinics. LN, lymph node. AJCC, American Joint Committee on Cancer. DCIS, ductal carcinoma *in situ*. LCIS, lobular carcinoma *in situ*. IDC, invasive ductal carcinoma. ILC, invasive lobular carcinoma.

Using the American Joint Committee on Cancer (AJCC) TNM staging system, 13.0% and 24.7% of 223 screening-detected breast cancers among urban women were stage 0 and stage I (respectively), with 3.0% and 24.6% among 431 rural cases. Screening detected significantly higher proportions of early-stage (stage 0 + I) breast cancer among both urban cases (37.7%) and rural cases (27.6%) than were detected in the clinic [21.5% of 475 cases, both P values < 0.001]. The proportions of tumor sizes <2 centimeters among both urban (51.6%) and rural cases (40.1%) were also significantly higher than those among clinic-detected cases (30.9%, both P values < 0.001). The proportion of cases with lymph node involvement identified by screening among urban women (24.2%) was significantly lower than that detected in the clinic (48.8%, P < 0.001), though there was no significant difference among rural women (18.3%) between the proportions of screening- and clinic-detected cases. The proportions of carcinoma *in situ* were 13.0% and 3.0% for urban cases and rural cases, respectively (Fig. 6).

Sensitivity analyses after excluding the unknown group for each abovementioned tumor characteristic gave results similar to the primary results: more cancer in early stage (46.2% and 38.8% of screening-detected urban and rural cancers, vs. only 23.4% of clinic-detected cancers), smaller tumors (62.5% and 66.3% of screening-detected urban and rural cancers had tumor size <2 centimeters, vs. 35.8% of clinic-detected cancers), less lymph node involvement (38.0% and 47.3% of screening-detected urban and rural cancers vs. 52.3% of clinic-detected cancers), and better tumor behavior (14.0% and 6.0% of screening-detected urban and rural cancers were carcinoma *in situ* vs. 3.6% of clinic-detected cancers).

**Figure 7:**
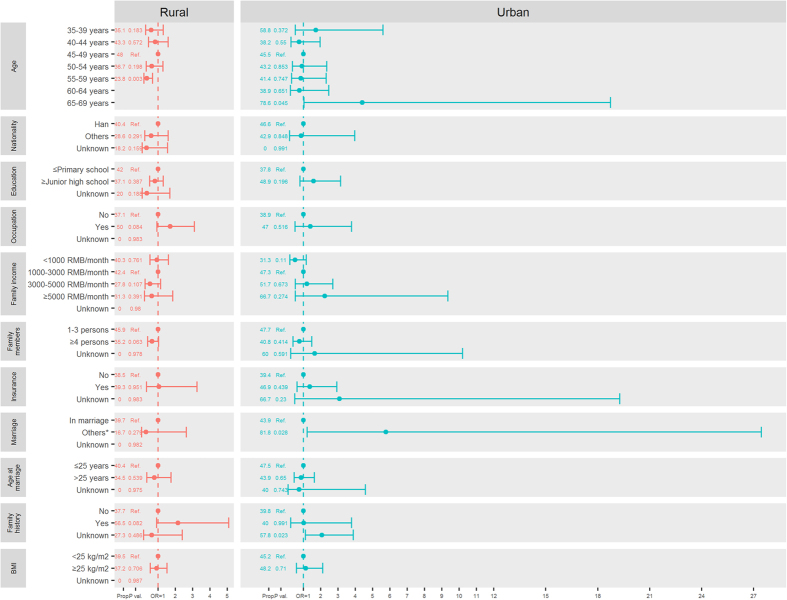
Distribution and odds ratios (OR) of early-stage breast cancer according to demographic characteristics from the Chinese National Breast Cancer Screening Program. Cases without clear TNM stage were excluded. *Included single, divorced, separated, and widow women.

After stratifying by demographic variables, a higher proportion of early-stage breast cancer was significantly associated with unmarried status for urban cases, and no factors were found to be significantly associated with a higher proportion of early-stage breast cancer among rural cases (Fig. 7).

## Discussion

The CNBCSP is the largest prospective population-based breast cancer screening cohort study ever in China. We not only achieved a breast cancer detection rate higher than the incidence rate of breast cancer reported by the Chinese cancer registry, but we also found more cancers with favorable tumor characteristics, i.e., earlier in stage, smaller in size, and with less lymph node involvement and better tumor behavior compared with clinic-detected cancers during the same period^11^. More important, based on more than 1.2 million Chinese women, we found several important differences between urban and rural women in screening parameters: detection rates, tumor characteristics, and patterns of cancer distribution. These results suggest that tailored screening schemes targeting specific populations are needed and would be expected to improve screening effectiveness.

As expected, the cancer detection rates in CNBCSP for Chinese urban women (0.6/1,000) and rural women (0.5/1,000) were much lower than those in western countries, including the United States (5.1/1,000 in the National Breast and Cervical Cancer Early Detection Program (NBCCEDP)^12^,^13^), United Kingdom (5.4/1,000 to 6.7/1,000 in the National Health Service Breast Screening Programme (NHS-BSP)^14^,^15^), and Canada (2.5/1,000 to 7.9/1,000 in the Canadian National Breast Screening Study (CNBSS)^16^,^17^). And the cancer detection rates in CNBCSP were even lower than that in the controls arm of CNBSS 2 (3.5/1,000) – the only study arm in which CBE was performed as the primary screening modality^17^. Several reasons, including the baseline incidence rates of breast cancer for different populations and different screening strategies conducted in different studies^1^,^2^,^12^,^13^,^14^,^15^,^16^,^17^, could lead to the lower detection rates in our study compared with previous studies. For example, in NBCCEDP, NHS-BSP and the intervention arm of CNBSS^12^,^13^,^14^,^15^,^16^,^17^, nearly all participants in screening programs receive MAM, while participants in CNBCSP are first screened by CBE, followed by MAM or BUS.

The most important result is that we not only found differences in preliminary effectiveness of breast cancer screening between Chinese urban and rural women, but also observed different factors associated with higher detection rates of breast cancer. These factors will be very useful in targeting high-risk populations for breast cancer screening. Certain factors, including older age at screening, family history of cancer, and obesity are known risk factors for breast cancer^18^,^19^,^20^. Other factors also reported here and in previous studies include lack of occupation or insurance (which indicate lower socioeconomic status and less access to breast-screening services)^21^,^22^,^23^. However, the only factor consistently associated with higher detection rates of breast cancer among both urban and rural women was older age at screening. Additionally, cancer detection rates among urban women tended to be more affected by obesity, while rates among rural women were more strongly affected by a family history of cancer. More interestingly, among Chinese urban women, non-obese women without employment or insurance seem to be at high risk for breast cancer. In addition to lower socioeconomic status and less chance to access breast-screening services, this subgroup of women without occupation or insurance would probably be the floating population in urban regions of China. Among Chinese rural women, unmarried women with a family history of cancer and high income should be considered a potential high-risk population. Among rural Chinese women, a high income may also suggest that the traditional Chinese high-fiber dietary pattern has begun to change to a more Western high protein-and-fat dietary pattern. Further studies are also needed to validate these results.

In addition, several previous hospital-based studies have suggested that breast tumor characteristics including stage, size, lymph node status, and histologic type are important prognostic factors^24^,^25^,^26^. Given more favorable tumor characteristics, we could expect our screening-detected breast cancer patients to have better outcomes than those with clinic-detected tumors. And we also found that among urban women, a family history of cancer was significantly associated with higher proportion of early-stage breast cancers. Therefore, future screening targeting these special populations would not only achieve a higher detection rate (as mentioned above), but could identify and treat more women with operable screening-detected early-stage cancer.

Another concern that should be addressed is the starting age (35 years) of screening in our program. Thirty-five years would be really too young to start regular screening according to previous screening trials conducted in western countries. However, in our study, we set the starting age of screening as 35 years based on the following three major considerations. First, according to age distribution of breast cancer from Chinese Cancer Registry, the starting high-risk age of breast cancer for both urban and rural women was 35 years old. Second, the mean age at diagnosis of breast cancer in China is considerably younger than that for western women^6^. Third, we set the age of starting screening as 35 years in our current study, it does not mean that we will recommend 35 years as the starting age of regular screening in the future Chinese guideline of breast cancer screening. In addition, according to the recommendations of the American Cancer Society, the suggested starting age for regular mammography screening was 40 in 2003^27^. Until 2015, the starting age for regular screening was changed to 45^28^. Therefore, based on the abovementioned considerations and the available guideline recommendations before the starting year of CNBCSP, we set the starting age of screening as 35 years in our current study.

Several limitations can be found in our study. First, due to lack of an unscreened control group, we cannot accurately determine the effectiveness of our screening program, despite having conducted several indirect comparisons (between detection rates of breast cancer in our program and incidence of breast cancer reported by the Chinese cancer registry over the same period; and between screening-detected cancers and clinic-detected cancers during the same period). Second, according to IARC reviews, there is inadequate evidence of mortality reduction based on CBE^29^. However, IARC also points out that there is sufficient evidence that tumors that were detected shifted toward an earlier stage^29^. The most important evidence came from another large developing country—India^30^. Therefore, ours would be first large population-based study of whether CBE should be introduced in future population-based screening programs in China. Third, due to our reliance on only one round of screening, our screening effectiveness was based solely on prevalence rather than incidence. Lastly, due to limited social resources, only selected breast cancer risk factors (such as family history of cancer and obesity) were investigated in our program. Therefore, we could not develop a comprehensive risk-assessment strategy based on known risk factors of breast cancer to identify population potential at high risk of breast cancer.

## Conclusions

The CNBCSP is the largest prospective population-based breast cancer screening cohort study ever in China and has achieved preliminary effectiveness among both urban and rural women. Between Chinese urban and rural women, we found several important differences in detection rates, tumor characteristics, and patterns of cancer distribution. In the future, feasible screening schemes targeting specific populations are needed and would be expected to achieve higher detection rates of breast cancer. Additional cost-effectiveness analyses are needed to evaluate the health-economic benefits of different screening methods.

## Methods

### Overview of the Chinese National Breast Cancer Screening Program

A total of 1,226,714 women aged 35–69 years (including 398,184 urban women and 828,530 rural women) were enrolled in the Chinese National Breast Cancer Screening Program (CNBCSP). Eligibility criteria included: (1) being of high-risk age for breast cancer according to the Chinese Cancer Registry for Chinese urban (age 35–69) and rural women (age 35–59)^31^, (2) no diagnosis of breast cancer before screening; and 3) having lived for ≥3 years in their local community. The screening program was conducted from March 2008 to November 2009 among Chinese urban women, and from July 2009 to December 2011 among Chinese rural women.

Before screening, the target population was selected from one or two urban communities with high incidence of breast cancer, were chosen by the provincial cancer registry according to local cancer registry data. Then a list of women in the target population was acquired from the local department of household registration from the Public Security Bureau or local residential committees. Archives including detailed information (name, person ID, age, and address) of the target population were established. Invitation letters with health education information were sent to all eligible women by the community staff, who also suggested a time for a screening appointment. The response rates of urban and rural women for breast cancer screening were 54.37% and 63.10%, respectively (Supplementary Table 6).

At the beginning of screening, after signing informed consent regarding all experimental protocols, all eligible women underwent a face-to-face interview to collect demographic data. Then trained physicians performed a clinical breast examination (CBE) on each participant. Urban women suspected of having cancer underwent mammography (MAM), and then underwent breast ultrasound (BUS) examination. However, MAM devices were not easily accessed due to limited medical resources in Chinese rural regions, and BUS was more available than MAM in these regions. Therefore, after undergoing CBE, rural women suspected of having cancer underwent BUS followed by MAM. This procedure is currently more suitable for women in Chinese rural regions according to the committee of experts of CNBCSP. Finally, needle aspiration biopsy or surgery was performed to confirm diagnosis in those with a highly suspected malignancy suggested by either MAM or BUS. One-year follow-up was conducted to validate negative results. The screening flowchart from CNBCSP is shown in Supplementary Fig. 2.

### Data collection

During the face-to-face interview, demographic data collected from all participants via structured questionnaire included participants’ age, marital status, age at marriage, nationality, educational level, occupation, number of family members living with participant, family income, health insurance, and family history of cancer. Body height and weight were measured by trained investigators. For detailed quality control measures of height and weight, please refer to our previously published paper^32^. And BMI calculated as weight in kilograms divided by the square of height in meters (kg/m^2^). Obesity was defined as BMI ≥25 kg/m^2^.

Data gathered during the CBE screening (included any signs of palpable lumps in the breast(s) or axilla, skin thickening or retraction, or nipple discharge or retraction) were noted and recorded on a structured form. Data from imaging (including mass characteristics, architectural distortion, asymmetric density, and calcification) were recorded on case report forms designed for BUS and MAM. If there was more than one lesion (multifocal) in a subject’s breast(s), the largest lesion was recorded. Final diagnostic results from CBE/BUS examinations were recoded categorically as: (1) normal, (2) abnormal benign, (3) suspicious malignancy, and (4) highly suggestive of a malignancy. MAM results were recorded according to the American College of Radiology Breast Imaging Reporting and Data System (BI-RADS) and categorized as: (0) additional imaging needed; (1) negative; (2) benign finding; (3) probably benign finding; (4) suspicious malignancy; and (5) highly suggestive of a malignancy^33^. For each examination, both (4) and (5) were considered positive, and other diagnostic results were considered negative.

The pathological examination was used as the reference standard for breast cancer diagnosis. For all breast cancers confirmed by pathological examination, clinical data on tumor characteristics (including tumor stage, tumor size, lymph node status, and histological type) were obtained from pathological reports. Tumor stage was defined according to the American Joint Committee on Cancer (AJCC) TNM staging system^34^. Stages 0 and I were considered early stage, and stages II, III, and IV were considered advanced stages in the analysis.

### Quality control

Various training courses and seminars based on the study protocol were conducted in a train-the-trainer model^35^. Briefly, in the first step, all provincial investigators, technicians, and physicians participating in the study were trained to learn the standardized epidemiological interview and screening procedures for CBE, BUS, and MAM. In the second step, the local investigators, technicians, and physicians participating in the study were trained by the provincial investigators, technicians, and physicians. Regular intensive training was also provided for local investigators, technicians, and physicians to ensure adherence to protocols.

Bilateral BUS assessments were performed by one or two technicians specializing in ultrasound, and bilateral mammograms were obtained using a full-field digital mammography system. For all imaging examinations, 5% of films were randomly selected for reassessment. Discrepancies between primary assessment and reassessment were resolved by a senior radiologist. For detailed quality control measures of mammography assessment, please refer to our previously published paper^36^.

All the examination data were double-entered and checked for consistency. Further comprehensive logic checking was conducted to remove logic errors between variables before analyses.

All the methods were in accordance with approved guidelines. All experimental protocols were approved by the Institutional Review Board of Tianjin Medical University Cancer Institute and Hospital, and informed consent was obtained from all subjects regarding all experimental protocols.

### Statistical analysis

The overall breast cancer detection rates and subgroup detection rates, stratified by demographic characteristics, were separately calculated for urban women and rural women. The ORs and their 95% CIs were calculated to evaluate the association between detection rates of breast cancer and demographic variables. Further stratified chi-squared tests were used to determine whether there was a cluster of factors associated with higher rates of breast cancer detection.

The clinical characteristics of screening-detected cancers were compared with those of clinic-detected cancers from the same period, obtained from a nation-wide multicenter retrospective clinical epidemiological study of female breast cancer in China^9^. After chi-squared tests the overall proportions of early-stage breast cancer and demographic subgroups were calculated for urban women and rural women.

After excluding missing values for all aforementioned analyses, sensitivity analyses were conducted to validate the primary results. Data analyses were conducted with SAS software version 9.3 and R 3.2.3. Two-sided P < 0.05 was considered statistically significant. Maps were created with the package ggplot2 (version 2.1.0) of R software (version 3.2.3).

## Additional Information

**How to cite this article**: Huang, Y. *et al*. Preliminary effectiveness of breast cancer screening among 1.22 million Chinese females and different cancer patterns between urban and rural women. *Sci. Rep.* **6**, 39459; doi: 10.1038/srep39459 (2016).

**Publisher's note:** Springer Nature remains neutral with regard to jurisdictional claims in published maps and institutional affiliations.

## Supplementary Material

Supplementary Information

## Figures and Tables

**Table 1:** Characteristics of participants in the Chinese National Breast Cancer Screening Program.

Variable	Urban women (N = 398184)		Rural women (N = 828530)	
	N	%	N	%
Age				
35–39 years	96771	24.3	208224	25.1
40–44 years	88461	22.2	206160	24.9
45–49 years	74393	18.7	189462	22.9
50–54 years	58133	14.6	113336	13.7
55–59 years	42036	10.6	111348	13.4
60–64 years	24067	6.0		
65–69 years	14323	3.6		
Nationality				
Han	384511	96.6	717684	86.6
Others	11784	3.0	82840	10.0
Unknown	1889	0.5	28006	3.4
Education				
≤Primary school	81504	20.5	375872	45.4
≥Junior high school	315729	79.3	430208	51.9
Unknown	951	0.2	22450	2.7
Occupation				
No	20068	5.0	647399	78.1
Yes	377259	94.7	170890	20.6
Unknown	857	0.2	10241	1.2
Family income				
<1000 Chinese RMB/month	76475	19.2	247694	29.9
1000–3000 Chinese RMB/month	212941	53.5	449748	54.3
3000–5000 Chinese RMB/month	74842	18.8	82309	9.9
≥5000 Chinese RMB/month	31305	7.9	29832	3.6
Unknown	2621	0.7	18947	2.3
Family members				
1–3 persons	269532	67.7	312811	37.8
≥4 persons	106233	26.7	501832	60.6
Unknown	22419	5.6	13887	1.7
Insurance				
No	55652	14.0	18734	2.3
Yes	320125	80.4	796375	96.1
Unknown	22407	5.6	13421	1.6
Marriage				
In marriage	381442	95.8	807725	97.5
Others^1^	16253	4.1	10220	1.2
Unknown	489	0.1	10585	1.3
Age at marriage				
≤25 years	279812	70.3	752078	90.8
>25 years	109960	27.6	64481	7.8
Unknown	8412	2.1	11971	1.4
Family history				
No	261861	65.8	763815	92.2
Yes	15952	4.0	42519	5.1
Unknown	120371	30.2	22196	2.7
Body mass index				
<25 kg/m^2^	310416	78.0	625374	75.5
≥25 kg/m^2^	87024	21.9	188227	22.7
Unknown	744	0.1	14929	1.8

^1^Included single, divorced, separated, and widowed women.
