# Supplementary material for: Preliminary effectiveness of breast cancer screening among 1.22 million Chinese females and different cancer patterns between urban and rural women
Source: Sci Rep. 2016 Dec 20;6:39459. doi: 10.1038/srep39459 (PMC5171648; doi:10.1038/srep39459)
Supplement: Supplementary Information [file srep39459-s1.doc]

**Supplementary Information**

**Preliminary effectiveness of breast cancer screening among 1.22 million Chinese females and different cancer patterns between urban and rural women**

Yubei Huang, Hongji Dai, Fengju Song, Haixin Li, Ye Yan, Zhenhua Yang, Zhaoxiang Ye, Sheng Zhang, Hong Liu, Yali Cao, Li Xiong, Yahong Luo, Tie Pan, Xiangjun Ma, Jie Wang, Xiuling Song, Ling Leng, Yeping Zhang, Jie Sun, Jialin Wang, Hengmin Ma, Lingzhi Kong, Zhenglong Lei, Yaogang Wang, Peishan, Wang, Jiali Han, Xishan Hao, and Kexin Chen


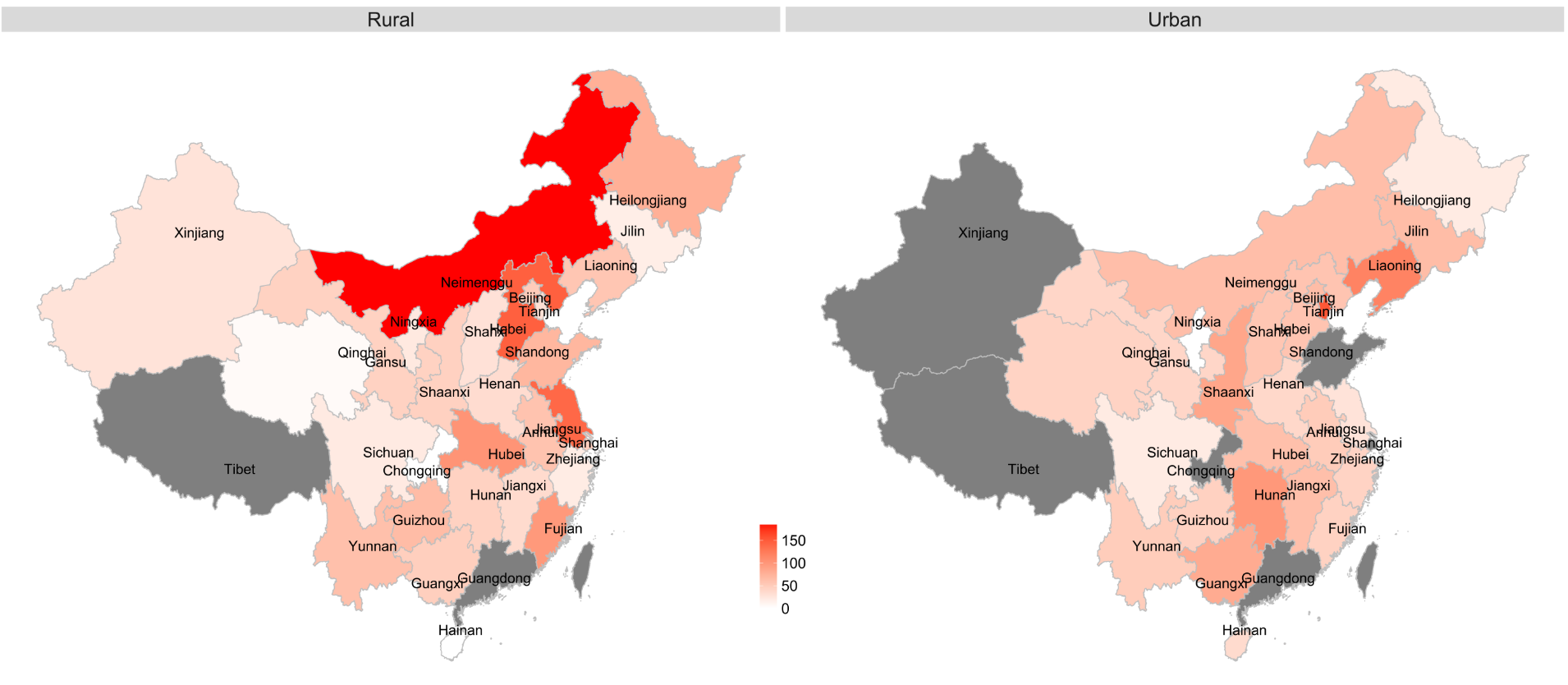


**Supplementary Figure 1**. **Provincial detection rates (1/10^5^) of breast cancer for urban and rural women from the Chinese National Breast Cancer Screening Program.** The maps were created by with the package ggplot2 (version 2.1.0. H. Wickham. ggplot2: Elegant Graphics for Data Analysis. Springer-Verlag, New York, 2009) of R software (version 3.2.5. R Core Team (2016). R: A language and environment for statistical computing. R Foundation for Statistical Computing, Vienna, Austria. URL https://www.R-project.org/).


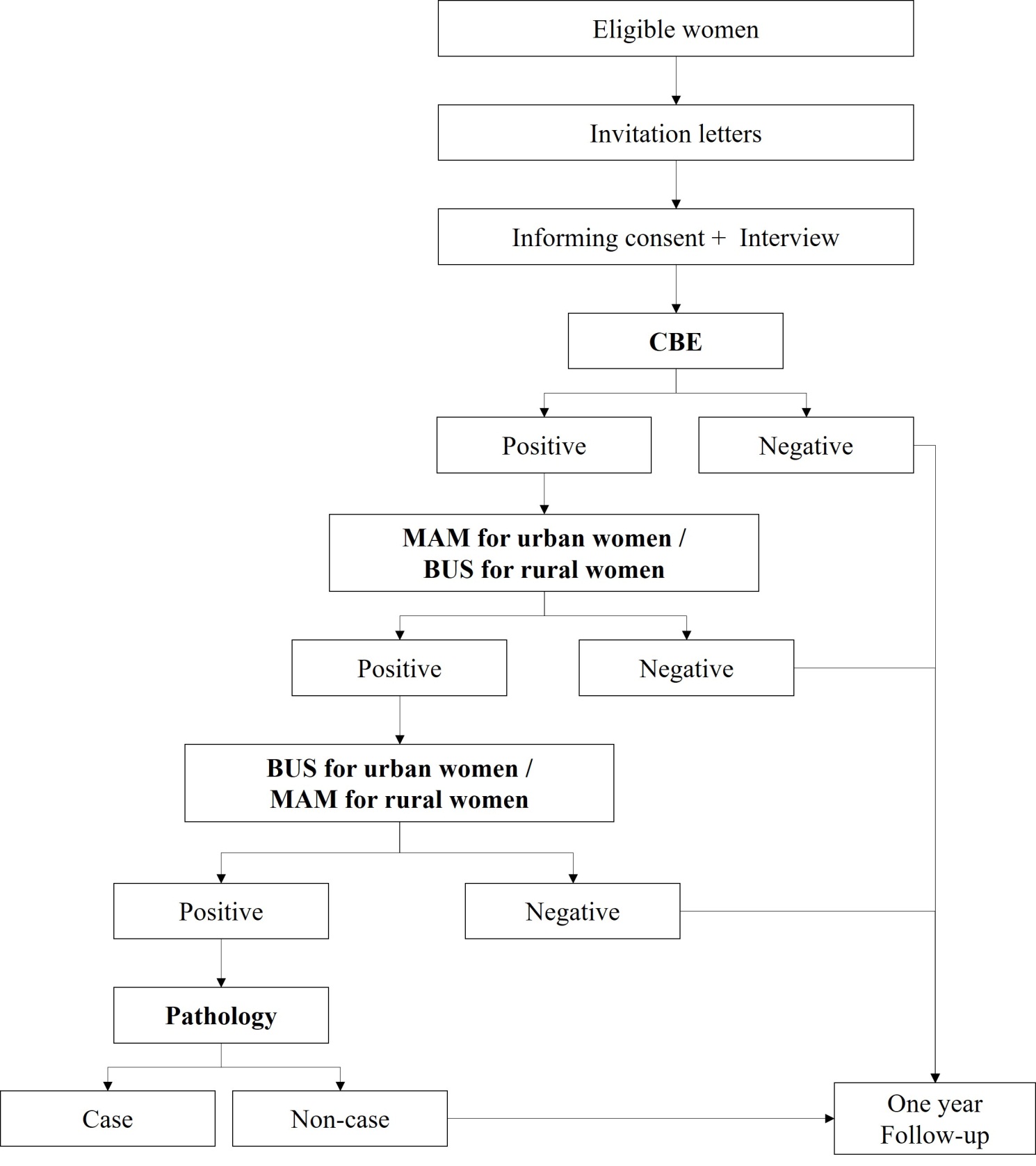


**Supplementary Figure 2.** Screening Flowcharts of the Chinese National Breast Cancer Screening Program for urban women (A), rural women (B).

| Variable | Urban (N=398,184) | | | Rural (N=828,530) | | |
| --- | --- | --- | --- | --- | --- | --- |
|  | Cases (DR,  1/1,000) | OR  (95%CI) | P value | Cases (DR,  1/1,000) | OR  (95%CI) | P value |
| Age |  |  |  |  |  |  |
| 35-39 yrs | 17 (0.18) | 1 |  | 59 (0.28) | 1 | 0.047 |
| 40-44 yrs | 40 (0.45) | 2.58 (1.46-4.54) | 0.001 | 82 (0.40) | 1.40 (1.00-1.96) | <0.001 |
| 45-49 yrs | 45 (0.60) | 3.44 (1.97-6.02) | <0.001 | 133 (0.70) | 2.48 (1.82-3.37) | <0.001 |
| 50-54 yrs | 46 (0.79) | 4.51 (2.58-7.86) | <0.001 | 73 (0.64) | 2.27 (1.61-3.21) | <0.001 |
| 55-59 yrs | 37 (0.88) | 5.01 (2.82-8.91) | <0.001 | 84 (0.75) | 2.66 (1.91-3.72) | <0.001 |
| 60-64 yrs | 22 (0.91) | 5.21 (2.76-9.81) | <0.001 |  |  |  |
| 65-69 yrs | 16 (1.12) | 6.36 (3.22-12.60) | <0.001 |  |  |  |
| Nationality |  |  |  |  |  |  |
| Han | 214 (0.56) | 0.82 (0.40-1.66) | 0.580 | 390 (0.54) | 1.55 (1.06-2.26) | 0.021 |
| Others | 8 (0.68) | 1 |  | 29 (0.35) | 1 |  |
| Unknown | 1 (0.53) | 0.78 (0.10-6.24) | 0.814 | 12 (0.43) | 1.22 (0.62-2.40) | 0.555 |
| Education |  |  |  |  |  |  |
| ≤Primary school | 57 (0.70) | 1 |  | 199 (0.53) | 1 |  |
| ≥Junior high school | 165 (0.52) | 0.75 (0.55-1.01) | 0.058 | 221 (0.51) | 0.97 (0.80-1.18) | 0.758 |
| Unknown | 1 (1.05) | 1.50 (0.21-10.87) | 0.686 | 11 (0.49) | 0.93 (0.50-1.70) | 0.802 |
| Occupation |  |  |  |  |  |  |
| No | 18 (0.90) | 1.65 (1.02-2.67) | 0.041 | 342 (0.53) | 1.07 (0.85-1.36) | 0.554 |
| Yes | 205 (0.54) | 1 |  | 84 (0.49) | 1 |  |
| Unknown | 0 (0.00) | - | 0.495 | 5 (0.49) | 0.99 (0.40-2.45) | 0.988 |
| Family income |  |  |  |  |  |  |
| <1000 RMB/month | 42 (0.55) | 1 |  | 101 (0.41) | 1 |  |
| 1000-3000 RMB/month | 130 (0.61) | 1.11 (0.78-1.57) | 0.551 | 247 (0.55) | 1.35 (1.07-1.70) | 0.012 |
| 3000-5000 RMB/month | 39 (0.52) | 0.95 (0.61-1.47) | 0.813 | 52 (0.63) | 1.55 (1.11-2.17) | 0.010 |
| ≥5000 RMB/month | 11 (0.35) | 0.64 (0.33-1.24) | 0.187 | 24 (0.80) | 1.97 (1.26-3.08) | 0.003 |
| Unknown | 1 (0.38) | 0.69 (0.10-5.05) | 0.719 | 7 (0.37) | 0.91 (0.42-1.95) | 0.801 |
| Residents |  |  |  |  |  |  |
| 1-3 persons | 159 (0.59) | 1 |  | 168 (0.54) | 1 |  |
| ≥4 persons | 58 (0.55) | 0.93 (0.69-1.25) | 0.614 | 256 (0.51) | 0.95 (0.78-1.15) | 0.604 |
| Unknown | 6 (0.27) | 0.45 (0.20-1.03) | 0.057 | 7 (0.50) | 0.94 (0.44-2.00) | 0.869 |
| Insurance |  |  |  |  |  |  |
| No | 45 (0.81) | 1.51 (1.09-2.10) | 0.013 | 14 (0.75) | 1.45 (0.85-2.47) | 0.171 |
| Yes | 171 (0.53) | 1 |  | 411 (0.52) | 1 |  |
| Unknown | 7 (0.31) | 0.58 (0.27-1.25) | 0.159 | 6 (0.45) | 0.60 (0.23-1.56) | 0.292 |
| Marriage |  |  |  |  |  |  |
| Married | 211 (0.55) | 1 |  | 416 (0.52) | 1 |  |
| Others^1^ | 12 (0.74) | 1.34 (0.75-2.39) | 0.330 | 10 (0.98) | 1.90 (1.02-3.56) | 0.045 |
| Unknown | 0 (0.00) | <0.001 | 0.994 | 5 (0.47) | 0.92 (0.38-2.22) | 0.848 |
| Age at marriage |  |  |  |  |  |  |
| ≤25 yrs | 146 (0.52) | 1 |  | 382 (0.51) | 1 |  |
| >25 yrs | 71 (0.65) | 1.24 (0.93-1.64) | 0.141 | 40 (0.62) | 1.22 (0.88-1.69) | 0.229 |
| Unknown | 6 (0.71) | 1.37 (0.61-3.10) | 0.453 | 9 (0.75) | 1.48 (0.76-2.87) | 0.245 |
| Family history |  |  |  |  |  |  |
| No | 136 (0.52) | 1 |  | 380 (0.50) | 1 |  |
| Yes | 12 (0.75) | 1.45 (0.80-2.62) | 0.218 | 36 (0.85) | 1.70 (1.21-2.40) | 0.002 |
| Unknown | 75 (0.62) | 1.20 (0.91-1.59) | 0.205 | 15 (0.68) | 1.36 (0.81-2.28) | 0.245 |
| BMI |  |  |  |  |  |  |
| ≤25 kg/m^2^ | 155 (0.50) | 1 |  | 328 (0.52) | 1 |  |
| >25 kg/m^2^ | 67 (0.77) | 1.54 (1.15-2.04) | 0.003 | 102 (0.54) | 1.03 (0.82-1.29) | 0.773 |
| Unknown | 1 (1.34) | 2.69 (0.15-12.01) | 0.323 | 1 (0.07) | 0.13 (0.01-0.56) | 0.039 |

**Supplementary Table 1. Detection rates of breast cancer according to demographic characteristics from the Chinese National Breast Cancer Screening Program.** DR, detection rate. 1, included single, divorced, separated, and widowed women.

| **Occupation** | **Insurance, N(%)** | | **P value** |
| --- | --- | --- | --- |
|  | **Yes** | **No** |  |
| Yes | 2151 (10.7) | 17870 (89.3) | <0.001 |
| No | 53275 (15.0) | 301360 (85.0) |  |

**Supplementary Table 2. Relationship between occupation and insurance among Chinese urban women.** Data with missing values for occupation, insurance, or body mass index were excluded.

| **Occupation/insurance** | **Obesity, N(%)** | | **P value** |
| --- | --- | --- | --- |
|  | **No** | **Yes** |  |
| No occupation, no insurance | 1686 (78.4) | 765 (21.6) | Ref. |
| No occupation, with insurance | 10683 (59.8) | 7187 (40.2) | <0.001 |
| With occupation, no insurance | 42434 (79.7) | 10841 (20.3) | 0.152 |
| With occupation, with insurance | 237649 (78.9) | 63711 (21.1) | 0.590 |

**Supplementary Table 3. Relationship between occupation/insurance and obesity (body mass index ≥25 kg/m^2^) among Chinese urban women.** Data with missing values for occupation, insurance, and body mass index were excluded.

| **Family income** | **Marriage, N(%)** | | **P value** |
| --- | --- | --- | --- |
|  | **In marriage** | **Others** |  |
| <1000 RMB/month | 237448 (98.1) | 4590 (1.9) | <0.001 |
| 1000-3000 RMB/month | 436698 (99.1) | 4184 (0.9) |  |
| 3000-5000 RMB/month | 78990 (99.2) | 604 (0.8) |  |
| ≥5000 RMB/month | 28728 (99.1) | 262 (0.9) |  |

**Supplementary Table 4**. Relationship between family income and marriage among Chinese rural women. Data with missing values for family income, marriage, and family history of cancer were excluded.

| **Family income/marriage** | **Family history of cancer, N(%)** | | **P value** |
| --- | --- | --- | --- |
|  | **No** | **Yes** |  |
| Low income, in marriage | 227759 (95.9) | 9689 (4.1) | <0.001 |
| Low income, not in marriage | 4254 (92.7) | 336 (7.3) | 0.039 |
| High income, in marriage | 512852 (94.2) | 31564 (5.8) | <0.001 |
| High income, not in marriage | 4623 (91.5) | 427 (8.5) | Ref. |

**Supplementary Table 5**. Relationship between family income/marriage and family history of cancer among Chinese rural women. Data with missing values for family income, marriage, and family history of cancer were excluded.

|  | Urban | Rural |
| --- | --- | --- |
| Target population | 1675365 | 2256101 |
| Invited population | 732359 | 1312956 |
| Coverage rate | 43.71% | 58.20% |
| Responded population | 398184 | 828530 |
| Response rate | 54.37% | 63.10% |

**Supplementary Table 6. Coverage rates and response rates of breast cancer screening for urban and rural women from the Chinese National Breast Cancer Screening Program.**
